# Morphological and spectroscopic analysis of snow and glacier algae and their parasitic fungi on different glaciers of Svalbard

**DOI:** 10.1038/s41598-021-01211-8

**Published:** 2021-11-08

**Authors:** Marta J. Fiołka, Nozomu Takeuchi, Weronika Sofińska-Chmiel, Sylwia Wójcik-Mieszawska, Tristram Irvine-Fynn, Arwyn Edwards

**Affiliations:** 1 Department of Immunobiology, Maria Curie-Skłodowska University, Lublin, Poland; 2 Department of Earth Sciences, Graduate School of Science, Chiba University, Chiba, Japan; 3 Analytical Laboratory, Maria Curie-Skłodowska University, Lublin, Poland; 4 Department of Geography and Earth Sciences, Aberystwyth University, Llandinam Building, Aberystwyth, SY23 3DB UK; 5 Institute of Biological, Rural and Environmental Sciences, Aberystwyth University, Cledwyn Building, Aberystwyth, SY23 3DA UK

**Keywords:** Biotechnology, Cell biology, Ecology, Microbiology

## Abstract

The results show the morphological analyses and spectroscopic studies of snow and glacier algae and their parasitic fungi in Svalbard (High Arctic). Fixed algal cells of two species, *Sanguina nivaloides* and *Ancylonema nordenskioeldii,* were imaged using light microscopy, scanning electron microscopy (SEM), and atomic force microscopy (AFM). Fluorescence microscopy using Calcofluor white stain supported the observations of parasitic fungi on the algal cells. Images in brightfield microscopy showed chytrid-like fungi penetrating the cells of both algal species. Parasites were found to colonize the cells of *A. nordenskioeldii* and hypnozygotes of *S. nivaloides*, while no fungi infected the cyst stages of *S. nivaloides*. The autofluorescence analysis revealed the ability of *S. nivaloides* to glow when excited with different wavelengths, while *A. nordenskioeldii* did not fluoresce. The hypnozygotes of *S. nivaloides* emitted brighter fluorescence than the cysts, and the most intense luminosity was observed in the UV range. The Fourier-transform infrared spectroscopy (FTIR) and energy-dispersive X-ray spectroscopy (EDS) spectroscopic analysis showed differences in the chemical composition between samples collected from three different sites. Samples dominated by cyst cells were characterized by the presence of an abundant polysaccharide envelope.

## Introduction

Svalbard is a wildlife-rich Arctic area with a series of islands in the northernmost territory of Norway. The adaptation of Arctic organisms to harsh climatic conditions and their mutual relations are a particularly interesting issue. Despite the prevailing harsh climatic conditions, the Svalbard Archipelago is regarded as the "hot spot" of the Arctic in microbiological research^1^. Melting snowpacks and glacier ice surfaces are ecosystems occupied by numerous psychrophilic organisms such as bacteria, algae, or fungi^2–5^. One of the typical phenomena in Svalbard is red snow, which is caused by blooming of red-pigmented snow algae. It also frequently occurs in polar and alpine regions worldwide^6,7^. The first observations of colored snow in the Svalbard Archipelago date back to the seventeenth century, and the first scientific studies confirming the presence of snow algae date back to 1840^8^. The red snow observed in Svalbard is the result of the formation of the resting stages of *Sanguina nivaloides* (*Chlamydomonas nivalis*; Chlorophyceae) living in the snow. The cells of the alga accumulate carotenoids, including astaxanthin and its derivatives, which dominate other pigments^9^. These cytoplasmic secondary pigments mask and protect the chloroplast from intense solar radiation.

On ablating glacier surfaces in Svalbard brown- or violet-colored ice has also been reported, and the phenomena ascribed to the the presence of *Ancylonema nordenskioeldii* microalgae (Zygnematales, Streptophyta)^10,11^. The metabolism of these algae is adjusted to temperatures close to 0 °C and high levels of solar irradiance in summer and intense sunlight. Blooms of algae cause striking and macroscopically visible discoloration of ice, and the color depends on the prevailing cellular pigmentation^12^. *A. nordenskioeldii* is a cosmopolitan species, as indicated by reports of their discovery on several polar and alpine glaciers: Greenland^13^, Alaska^14^, Chile^15^, Himalayas^16^ or Maritime Antarctica^17^.

Chytrids are zoosporic fungi playing an ecological role in aquatic ecosystems. However, they are insufficiently investigated. The term parasite-host relationship between snow algae and fungus is fully justified in this case. Chytrids are often considered host-specific parasites^18^. However, this specificity has never been fully elucidated. Some parasitic fungi are known to have a narrow host range, while the host range of others can be very wide^19^.

In the Arctic marine environments, Chytridiomycota are the dominant fungi^20–25^. This group of the Kingdom Fungi is characterized by production of zoospores, otherwise known as motile cells, which have a single posterior flagellar structure. These flagellate gametes are reproductive cells with flagella allowing them to swim^26^. Generally, fungi do not have flagella, with few exceptions, which suggests that other fungi have lost this feature at some point in the evolutionary process^27^. This group with motile stages includes microscopic species, and most of them are associated with the environment of freshwater waters or moist soils. These organisms are mostly parasites of algae and animals and live on organic debris. Chytrids have chitin strengthening the cell walls. An exception is one subgroup (Hyphochytrids) that additionally has cellulose, which is a unique feature of fungi. Thus, the presence of chitin is an important characteristic defining these fungi.

Since little information known about snow algae and their parasitic fungi in Svalbard (and elsewhere), we undertook an analysis of these hosts and their parasites. In this study, we describe the cell morphology of the two major snow and glacier algal species and the associated parasitic fungi found in Svalbard and discuss the infectivity of the fungi in relation to the different morphological types of algal cells.

## Materials and methods

### Samples

**Figure 1:**
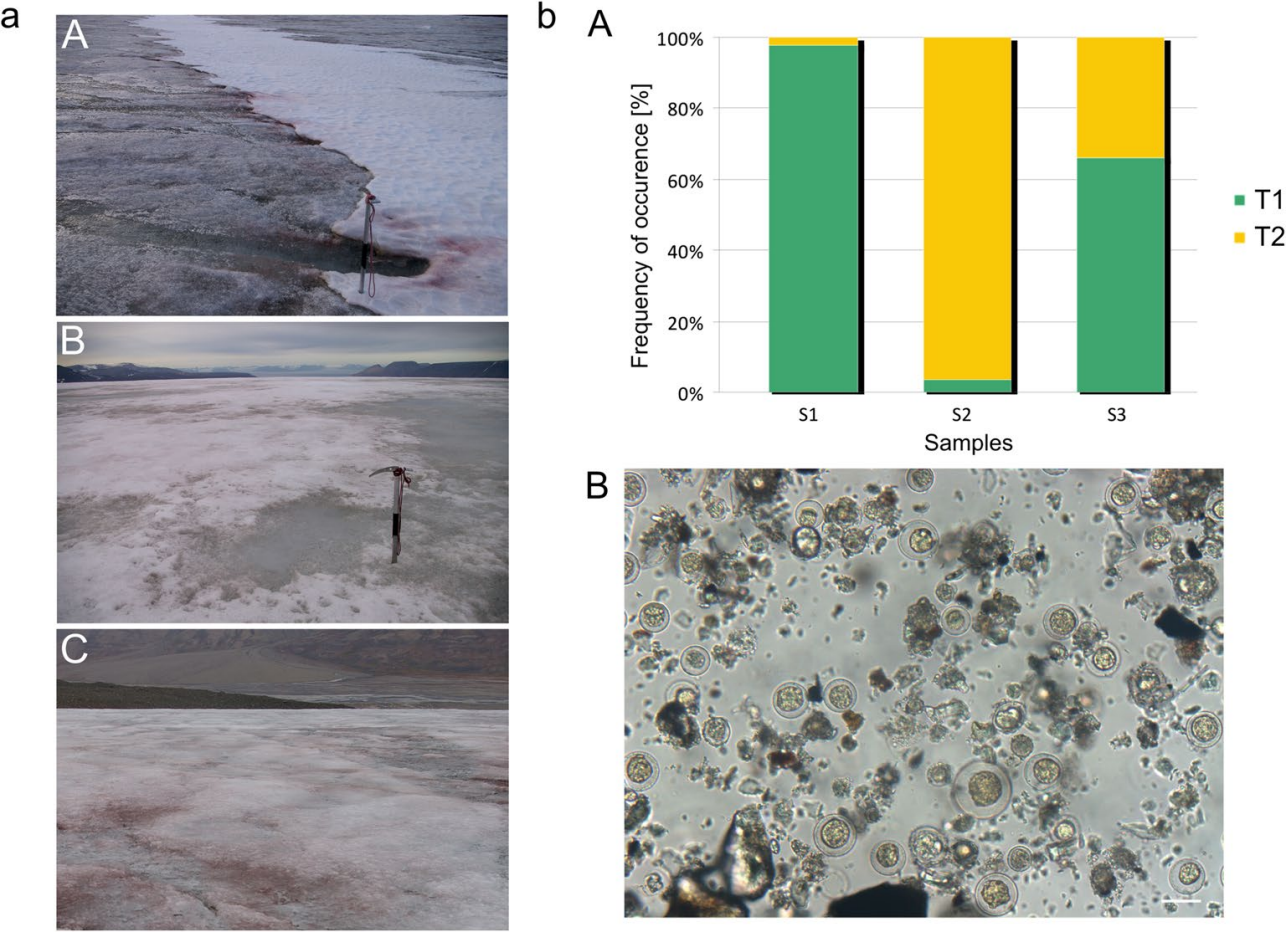
**(a)** Red snow and ice sampling sites in Svalbard: A—Longyearbreen Glacier, B—Upper Foxfonna Glacier (elevation 789 m), C—Lower Foxfonna Glacier (elevation 609 m). **(b)** A—Abundances of two morphological forms of *S. nivaloides*: T1—cysts, and T2—hypnozygotes in three sites: S1 (A), S2 (B), S3 (C); B—microscopic image of fixed algal cells from Longyearbreen Glacier. Scale: 20 µm. Pictures were taken by Nozomu Takeuchi.

Red snow/ice samples collected from Longyearbreen and Foxfonna Glaciers of Svalbard (Norway) were analyzed in this study. The samples were obtained with a stainless-steel scoop (1–2 cm in depth), melted, and preserved in a 3% formalin solution in sterile 30-ml polyethylene bottles. The locations of the sampling sites, and the associated pH and electrical conductivity are shown in Table 1. Images of red snow on all three glacier sites are presented in Fig. 1a.

**Table 1:** Locations of the sampling sites with pH and electrical conductivity (EC).

Site	Glacier	Position	Elevation (m)	pH	EC (µS/cm)	Collection date
S1	Longyearbreen	N78.17579, E15.49680	417 (m)	6.44	4.24	20.08.2011
S2	Upper Foxfonna	N78.12888, E16.17965	789 (m)	6.36	2.13	23.08.2011
S3	Lower Foxfonna	N78.14030, E16.15789	609 (m)	6.36	4.33	22.08.2011

### Light microscopy

Brightfield, Extended Depth of Field (EDF) microscopy, and Differential Interference Contrast (DIC) microscopy techniques were used to visualize the algal and fungal cells^28^. Two microlitres of the cell suspension in formalin were placed onto a microscope slide and photographed in brightfield microscopy with the use of immersion. The algal and fungal cells were observed under a Zeiss Axiovert 40CFL Carl Zeiss (Germany) light microscope. In the EDF microscopy, the microscope generates a series of multifocal images and finally produces an extended depth of field (EDF) image within a short time. EDF microscopic images of algal cells were taken using the MA200 Nikon optical microscope (Japan) with inverted optics equipped with a confocal attachment, which works with three lasers. DIC images of fungal cells were taken using the MA200 Nikon microscope. The contrast enhancing DIC technique for observation of microorganisms yields a three-dimensional object effect.

### Fluorescence analysis of algal and fungal cells

A Zeiss Axiovert 40CFL light microscope (Carl Zeiss, Germany) was used for imaging algal cells. The two types of snow algal cells were observed using brightfield microscopy and upon excitation at a 365 nm wavelength (Filter Set 02; Carl Zeiss) (blue light). Approximately 600 cells from each sample were analyzed. Images were recorded at 40 × magnification. The level of emitted fluorescence was measured with ImageJ and the image was converted to an 8- bit grayscale microscopic image to obtain five-digit fluorescence values. The results were expressed in Relative Fluorescence Units (RFU). RFU is the sum of the intensity and luminosity of all pixels measured in the microscopic image. The analyses involved 600 cells of each form of *S. nivaloides*. In addition, the cells were observed with the use of three filters with different excitation wavelengths: 365 nm (Filter Set 02, blue light), 470 nm (Filter Set 13) (green light), and 546 nm (Filter Set 15) (red light)^28^. The aim of the observation was to compare the luminosity of the algal and fungal cells.

### Analysis of algal and fungal cell fluorescence after staining with fluorochromes

The cells were stained with the Calcofluor white fluorochrome (CW) (Fluka, Germany), which binds to chitin in the fungal cell wall^29,30^ and cellulose in the algal cell wall^31^. A CW solution (3 drops of 10% KOH and 3 drops of 0.1% CW) was added in a proportion 1:2 to a suspension of algal cells and fungi and incubated for 10 min in the dark. Next, 2-µl samples were applied to a microscope slide and imaged with immersion at 450 nm. CW stains fresh samples and formalin- or glutaraldehyde-fixed samples. The cellulose wall of some forms of algae binds the dye and emits blue fluorescence^28^. Chytrid fungi at different stages of the life cycle (attached zoospores, spores, and empty spores) can be colored with this fluorochrome as well^18,32^.

Propidium iodide (PI) was used to stain *S. nivaloides*. This fluorochrome is a red fluorescent dye that contrasts with nuclei and chromosomes. PI does not penetrate living cells but enters cells with damaged cell membranes; therefore, it is considered an indicator of membrane integrity^33^. The algal cell suspension was mixed with an aqueous dye solution in a 1:2 volume ratio and incubated for 30 min at 37 °C in the dark. The cells were imaged in brightfield microscopy with the Zeiss Axiovert 40CFL light microscope.

### SEM of algal cells

Scanning electron microscopy (SEM) was used to visualize the algal and fungal cells. The cells were washed with 0.1 M phosphate buffer, pH 7.0, followed by fixation in 4% glutaraldehyde. After 4 h of incubation at room temperature, the cells were washed again in the same buffer, suspended in a 1% OsO_4_ solution, and incubated for 30 min. Dehydration was carried out using increasing concentrations of acetone (successively 30%, 50%, 70% and twice 100% for 30 min). The cell suspension was placed on microscope stages covered with carbon discs. The samples were then dried on silica gel in a desiccator for 24 h. The silica gel was previously prepared by incandescence in an oven at 121 °C for 2 h. The surface of the tables with algal cells was gold coated with a K550X sputtering machine (Quorum Technologies, United Kingdom). Samples with algal cells were then analyzed using a Tescan Vega 3 scanning electron microscope (Tescan Orsay Holding, Czech Republic)^28^. The *A. nordenskioeldii* cells were not dried, but when samples immersed in acetone were applied to the carbon discs, they were directly inserted into the vacuum chamber of the Quanta 3D FEG high-resolution scanning electron–ion microscope (FEI Company, Hillsboro, USA) and immediately analyzed to minimize the negative effects of drying.

### AFM analysis of algal cells

Algal cells prepared as for the SEM analysis (as above) in 100% acetone were placed on a mica disc and analyzed using atomic force microscopy (AFM). The cell surface was characterized using a NanoScope V AFM (Bruker, Vecco Instruments Inc., Billerica, MA, USA) in the Peak-Force Quantitative Nanomechanical Mapping Mode and NanoScope 8.15 software. The nominal spring rate of the RTESPA probe (Bruker, Billerica, MA, USA) (silicone tip on nitride lever) was 40 N/m.

### FTIR spectroscopy

The Fourier-transfer infrared spectroscopy (FTIR) method is based on absorption of radiation associated with the excitation of oscillating levels of molecules. This analysis can provide valuable information on the structure of organic compounds. The method is based on the characteristics of the absorption bands for selected functional groups of organic compounds. FTIR-ATR spectroscopy was used to analyze and identify compounds in the algal cell suspension and sample supernatants. The FTIR analyses were performed in a FTIR Nicolet 8700 spectrometer (Thermo Scientific, USA) and the ATR technique with a diamond crystal in the range of 4000–400 cm^−1^ wavenumbers and spectral resolution of 4 cm^−1^. Spectra were recorded from material fixed in a 3% aqueous formaldehyde solution. The sample was applied directly onto the diamond crystal of the ATR cap at room temperature. Additionally, the spectrum of a 3% formalin solution was analyzed and finally subtracted from the spectra obtained. The spectra were normalized.

### SEM/energy dispersive X-ray spectroscopy (EDX)

The extracellular fraction of the algal samples from the three sites in Svalbard was analyzed by XPS. The analyses were performed for five selected points of each sample and the arithmetic mean was calculated. A 1 mL volume of supernatant obtained after the centrifugation of the algal suspension for 15 min at 3000 rpm was frozen at −20 °C and then lyophilized to remove the formalin solution. The obtained powder was imaged by SEM and analyzed to determine its elemental composition. The samples were placed on aluminium microscope stages and transferred to the microscope chamber. The thickness of the samples prevented recording the Al signal from the stage. The microscope system was equipped with an EDAX spectrometer (EDS). The EDS measurements were performed with 30 kV beam energy. Five different points were analyzed for each sample. The elemental composition expressed by the atomic concentration of the test points was calculated by means of EDAX.

### X-ray photoelectron spectroscopy (XPS)

The algal suspension supernatant in a 3% formalin solution was frozen at −20 °C and freeze-dried to remove formalin. The powder obtained after lyophilization was examined by X-ray photoelectron spectroscopy to identify chemical bonds. The XPS analyses were performed using a multi-chamber UHV system (PREVAC). The spectra were acquired using a Scienta SAX-100 X-ray source (Al Kα, 1486.6 eV, 0.8 eV band) equipped with an XM 650 X-ray monochromator (0.2 eV band) and a Scienta R4000 hemispherical electron analyzer. The transition energy of the analyzer was set to 200 eV for the measurement of spectra (500 meV step) and 50 eV for the regions (high-resolution spectra) with a 50 meV step. The pressure in the analytical chamber during the collection of the spectra did not exceed 2 × 10^–8^ mbar. Samples from site 1 and site 3 were compared, as these samples comprised many cyst cells characterized by a polysaccharide envelope and no infections with the parasitic fungus.

### Statistical analysis

Statistical analysis of the algal cell fluorescence was performed using the Statistica program and the ANOVA Kruskal–Wallis test for nonparametric analysis; Kruskal–Wallis, H (3, N = 1030) = 60.9958; p = 0.000.

## Results

### Occurrence and size of algal cells

The samples contained mainly two morphological stages of *Sanguina* spp.: Type 1—roundish with a mucous envelope and Type 2—rosette-shaped cells. Types 1 and 2 are likely to be the cysts and hypnozygotes of this species, respectively^28^. The abundances of each type are shown in Fig. 1bA. The number of cells of the different types varied considerably between the sites. Type 1 cells dominated in site 1 and constituted more than 97%, whereas Type 2 cells with a similar percentage dominated in site 2. In turn, 60% of Type 1 cells and the remaining percentage of Type 2 were found in site 3 (Fig. 1bA). Figure 1bB shows a microscopic image of a sample from site 1 dominated by Type 1 cells.

**Figure 2:**
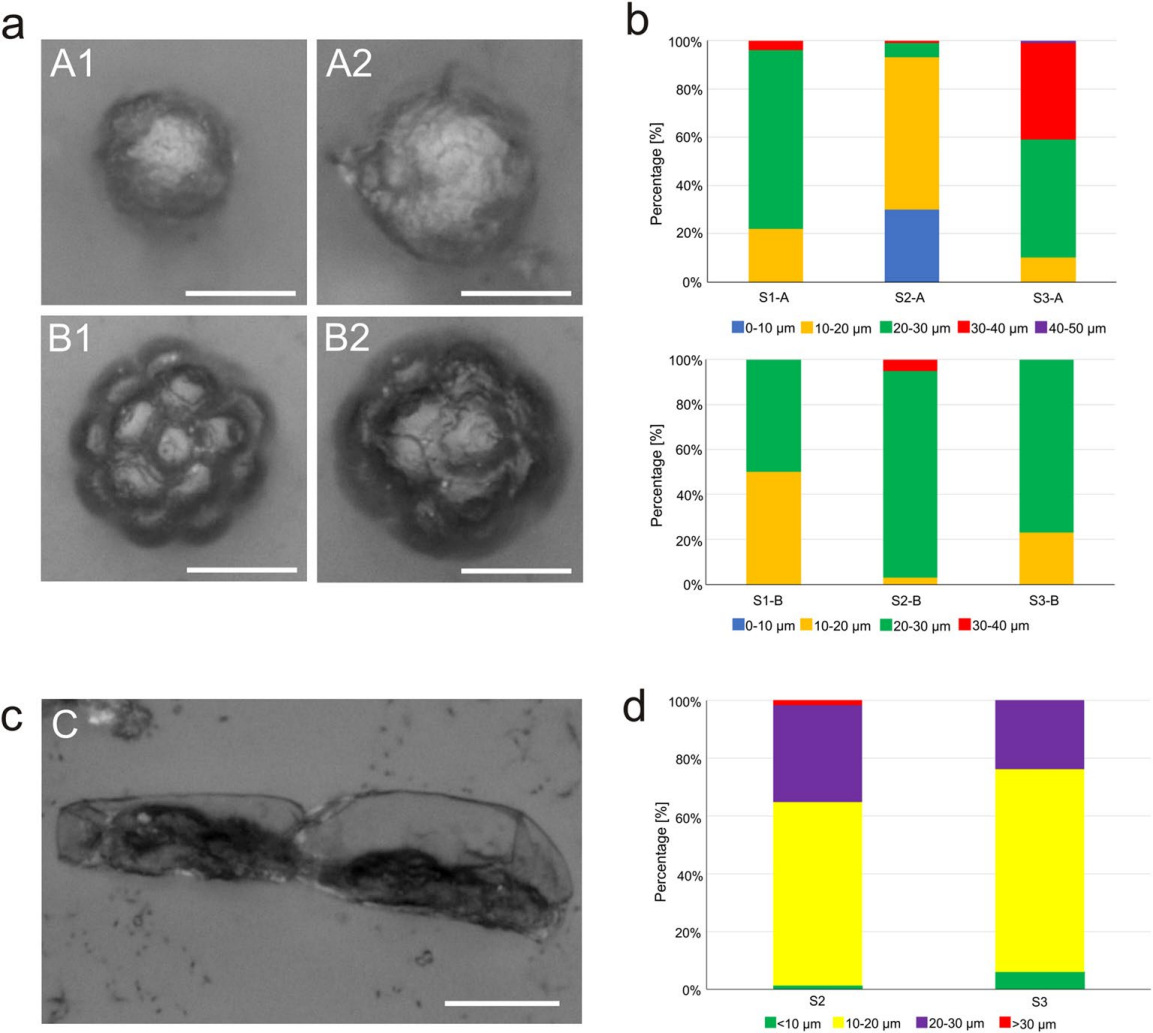
**(a)** EDF images of fixed *S. nivaloides*: smooth cysts (A1, A2) and rosette hypnozygotes (B1, B2); **(b)** cell size class abundances: cysts (top panel) and hypnozygotes (bottom panel) from three sites in Svalbard: S1, S2, S3; **(c)** EDF image of fixed *A. nordenskioeldii*; **(d)** cell size classes of *A. nordenskioeldii* from S2 and S3. Scale: 10 µm.

Figure 2a shows EDF images of algal cysts and hypnozygotes. Cysts in the size (diameter) range of 20–30 µm were dominant in site 1, and the dominant cysts in site 2 had a size of 10–20 µm. In site 3, as many as 49% of cells were in the range of 20–30 µm and 40% had a diameter of 30–40 µm (Fig. 2b, top graph). Hypnozygotes with a size in the range of 20–30 µm were most dominant in all the three sites and accounted for 50% in site 1.92% in site 2, and 77% in site 3 (Fig. 2b, bottom graph).

The samples from sites 2 and 3 also contained *A. nordenskioeldii*, which were visible as chains of elongated cells (Fig. 2c). Cells with a length of 10–20 µm accounted for almost 64% in site 2 and 70% in site 3. Cells in the 20–30 µm length range accounted for 33.5% in site 2 and 23.9% in site 3. Cells longer than 30 µm were found only in site 2, but they accounted for a small percentage of 1.73% (Fig. 2d). The observed width of all *A. nordenskioeldii* cells was about 9 µm. No *A. nordenskioeldii* algal cells were observed in the samples from site 1. This species constituted 48% of all cells in sample 2 and 24% in sample 3.

### Imaging of *S. nivaloides* cells using brightfield microscopy, EDF, AFM, SEM, and DIC

**Figure 3:**
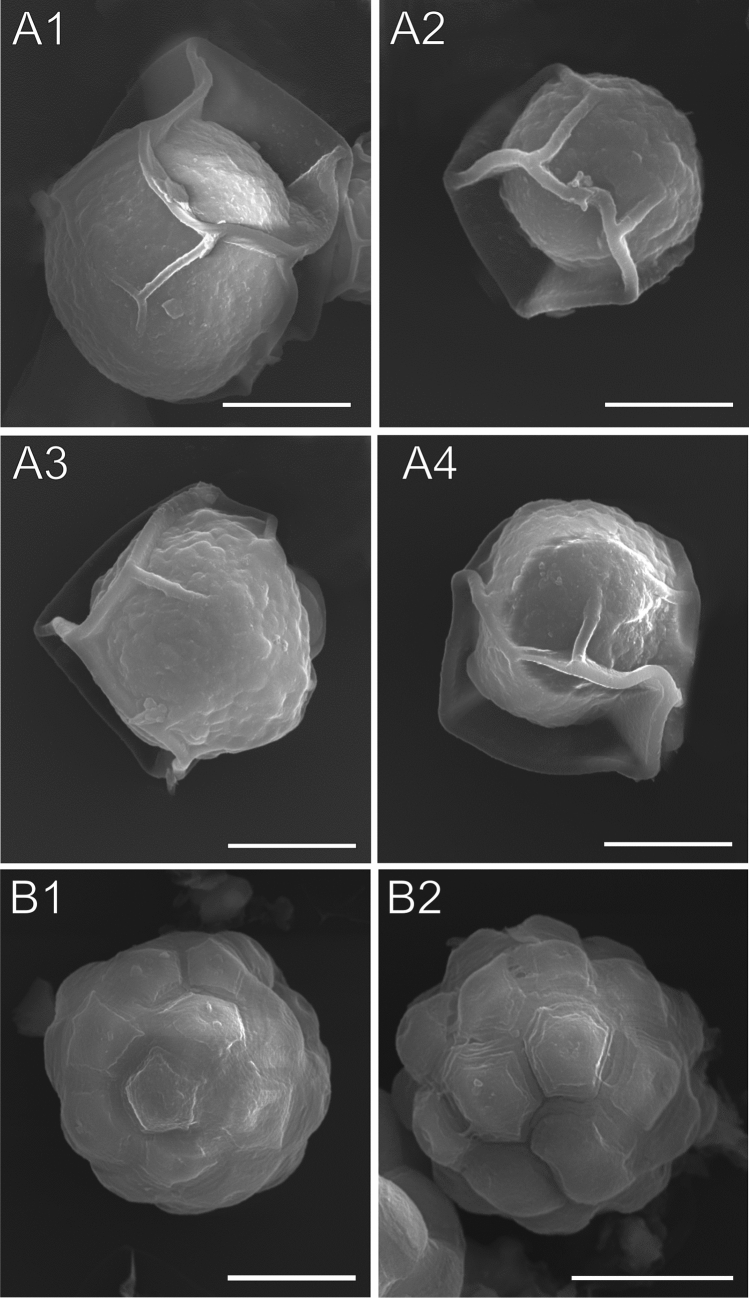
SEM images of fixed *S. nivaloides* from Longyearbreen Glacier: A1-A4—cysts, B1, B2—hypnozygotes. The scale bar corresponds to 10 µm.

**Figure 4:**
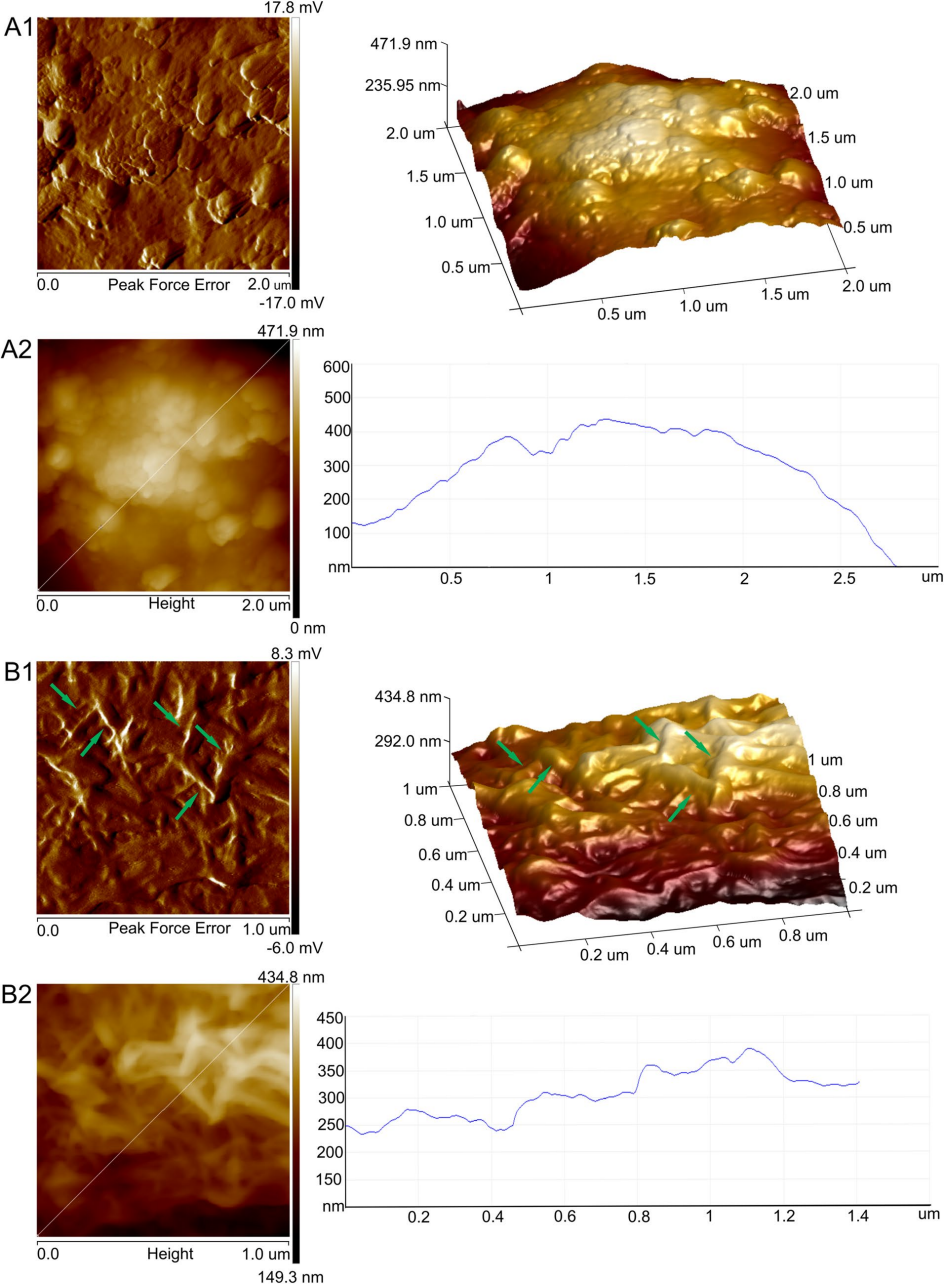
AFM images of the fixed *S. nivaloides* cyst and hypnozygote surface: peak force error images of surface fragments of the cyst (A1) and the hypnozygote (B1); 3D image of the cyst—right panel of A1 and the hypnozygote—right panel of B1; height images of the cyst (A2) and the hypnozygote (B2); the height profiles are presented in the right panel of A2 and B2. Cellulose fibers are pointed by green arrows.

The cells of *S. nivaloides* snow algae were imaged by EDF (Fig. 2a). Type 1—cyst cells, had a slightly rough surface, while Type 2 cells—hypnozygotes, had a surface with symmetrically arranged cone-shaped raised areas with a flattened top. The SEM imaging showed that Type 1 cells had delicate mucilage envelopes on the surface, while the apexes of cones in Type 2 cells appeared as a pentagonal disc (Fig. 3). Additionally, there were some algal cells differed significantly from the two types. They were characterized by a wrinkled surface without any coating and large sizes, compared to the typical cells of snow algae. The AFM analysis of both cell types confirmed the slight surface roughness of Type 1 cells (Fig. 4). Interestingly, intersecting cellulose fibers were visible on the surface of Type 2 cells. The network of cellulose fibers was visible in both the 2D and 3D images (Fig. 4B1 indicated with green arrows).

### *A. nordenskioeldii* cells imaging using microscopic techniques

**Figure 5:**
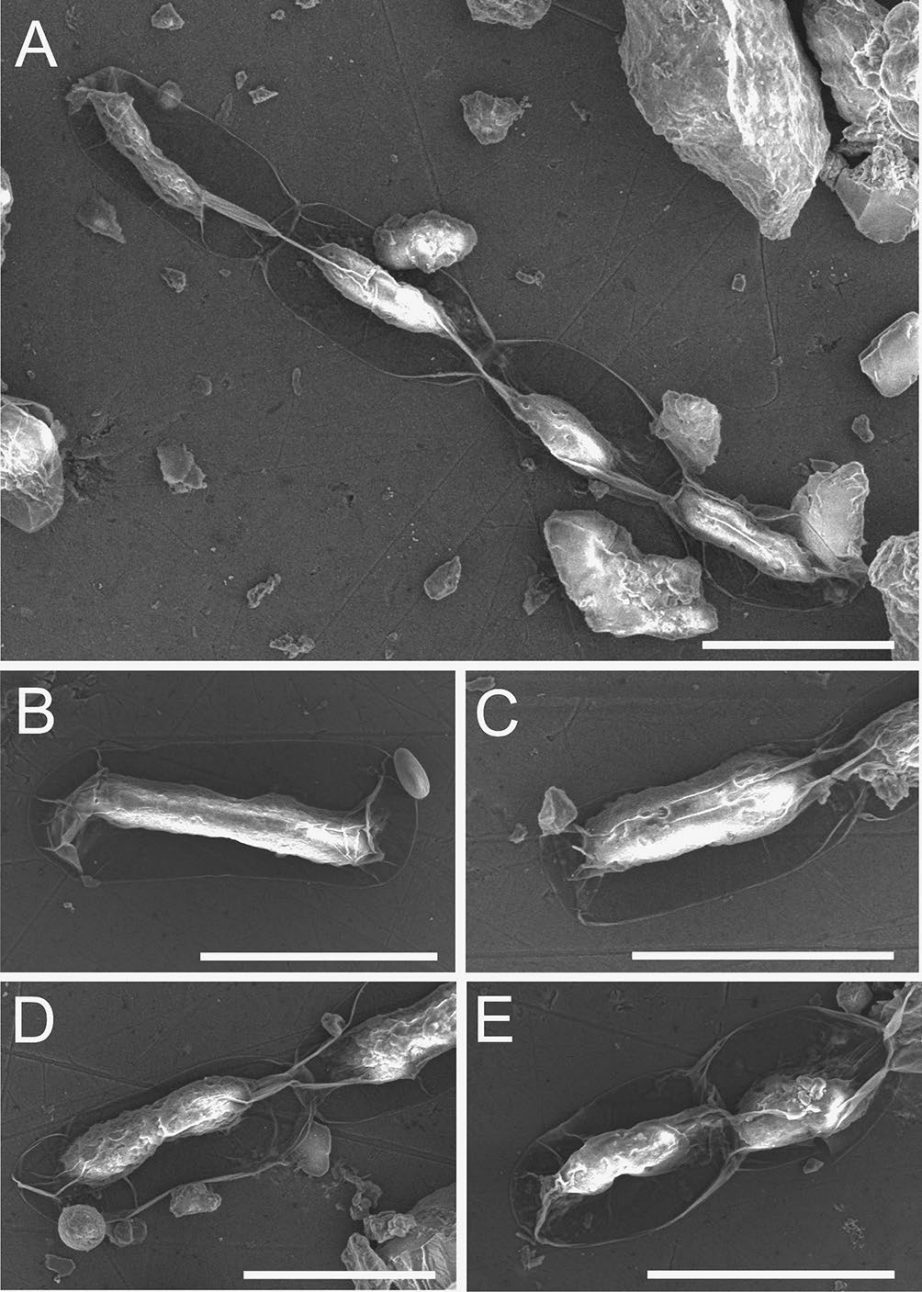
SEM images of fixed *A. nordenskioeldii* from Upper Foxfonna Glacier (elevation 789 m): A—chain of connected cells B-E—image of single and doublet cells; the cell wall is very thin, delicate, and shrinks easily. The images were taken immediately after placing the samples in the acetone solution in the microscope chamber to minimize the negative effects of drying. The scale bar corresponds to 20 µm.

**Figure 6:**
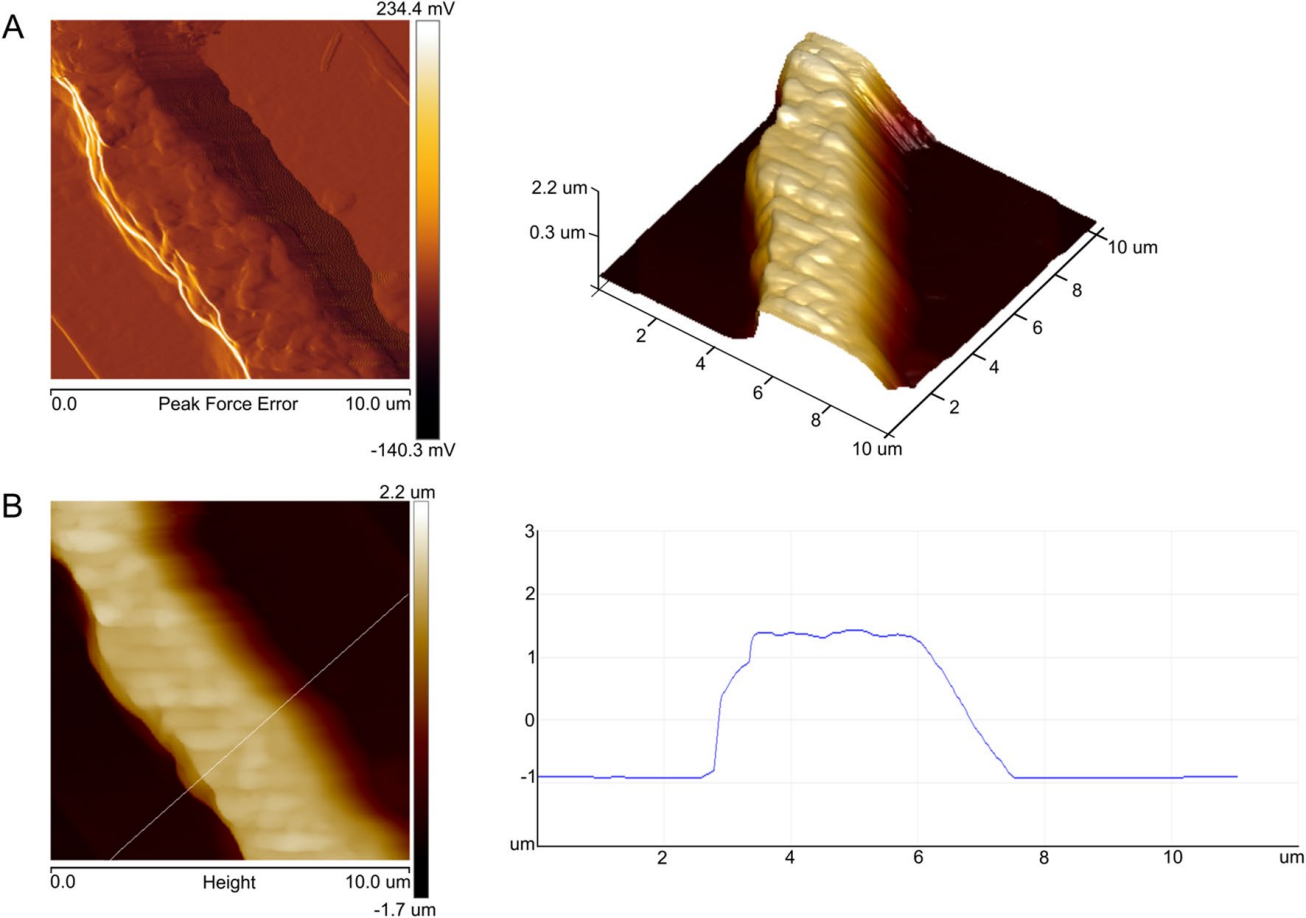
AFM images of fixed *A. nordenskioeldii* cells from Upper Foxfonna Glacier (elevation 789 m), A—peak force error images of a fragment of the fungal cell surface, 3D image—right panel; B—height image of the algal cell surface; the height profile is presented in the right panel.

The images of *A. nordenskioeldii* obtained by SEM correspond with the DIC photos. The SEM pictures show 20–30 µm long *A. nordenskioeldii* cells forming short filaments (Fig. 5). The cells are characterized by a clearly convex central part and a transparent envelope formed by a thin cell wall. The AFM analysis showed a regular and symmetrical shape of the algal cells (Fig. 6). There were slight folds on the cell surface, probably caused by the drying process. The length of the analyzed cells was 10–30 µm and the accompanying width was approx. 8.5–9.5 µm.

### Analysis of algal cell fluorescence

**Figure 7:**
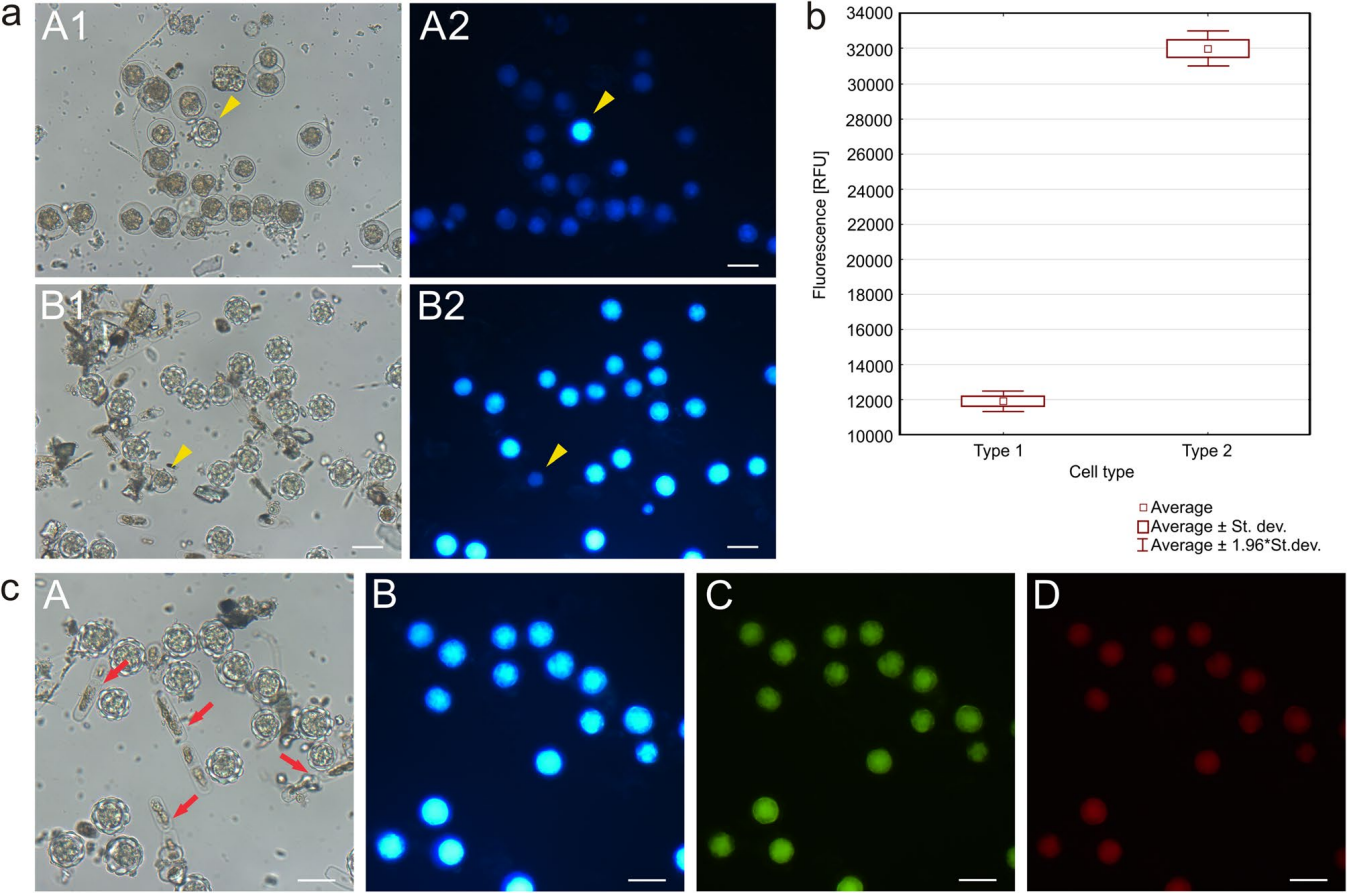
Fluorescence of fixed (pigment-free) algal cells from Svalbard: **(a)** Microscopic image of algal cells in brightfield microscopy (A1 from Longyearbreen Glacier, B1 from Upper Foxfonna Glacier (elevation 789 m) and after excitation with 365-nm wavelength light (A2, B2 as above); **(b)** fluorescence of cysts (Type 1) and hypnozygotes (Type 2) expressed in fluorescence units (RFU); **(c)** microscopic images of *S. nivaloides* hypnozygotes and *A. nordenskioeldii* cells (marked by red arrows) (from site 2) observed in brightfield microscopy (A) and light with a wavelength of 365 nm—blue, (B), 470 nm—green (C), and 546 nm—red. Hypnozygotes show the strongest fluorescence under the blue light, but no glow of *A. nordenskioeldii* cells was observed. The scale bar corresponds to 20 µm.

Analysis of the fluorescence of the algal cells after excitation with 365-nm wavelength light revealed that the Type 2 *S. nivaloides* cells fluoresced more than the Type 1 cells. The rosette-shaped cells emitted intense blue light, while the Type 1 cells showed a dim fluorescence without the characteristic luminosity (Fig. 7a). The fluorescence values largely differed between the two groups of types, as shown in the graph in Fig. 7b. Samples of Type 2 algal cells with fungal cells were analyzed to determine their fluorescence using filters with three different emission wavelengths: 365 nm (blue), 470 nm (green), and 546 nm (red). The fluorescence of the algal cells was the brightest in blue, weaker in green, and the weakest in red, likewise algal cells in Alaska characterized previously^28^. The *A. nordenskioeldii* cells indicated by red arrows showed no fluorescence (Fig. 7c).

### Microscopic analysis of algal and fungal cell

**Figure 8:**
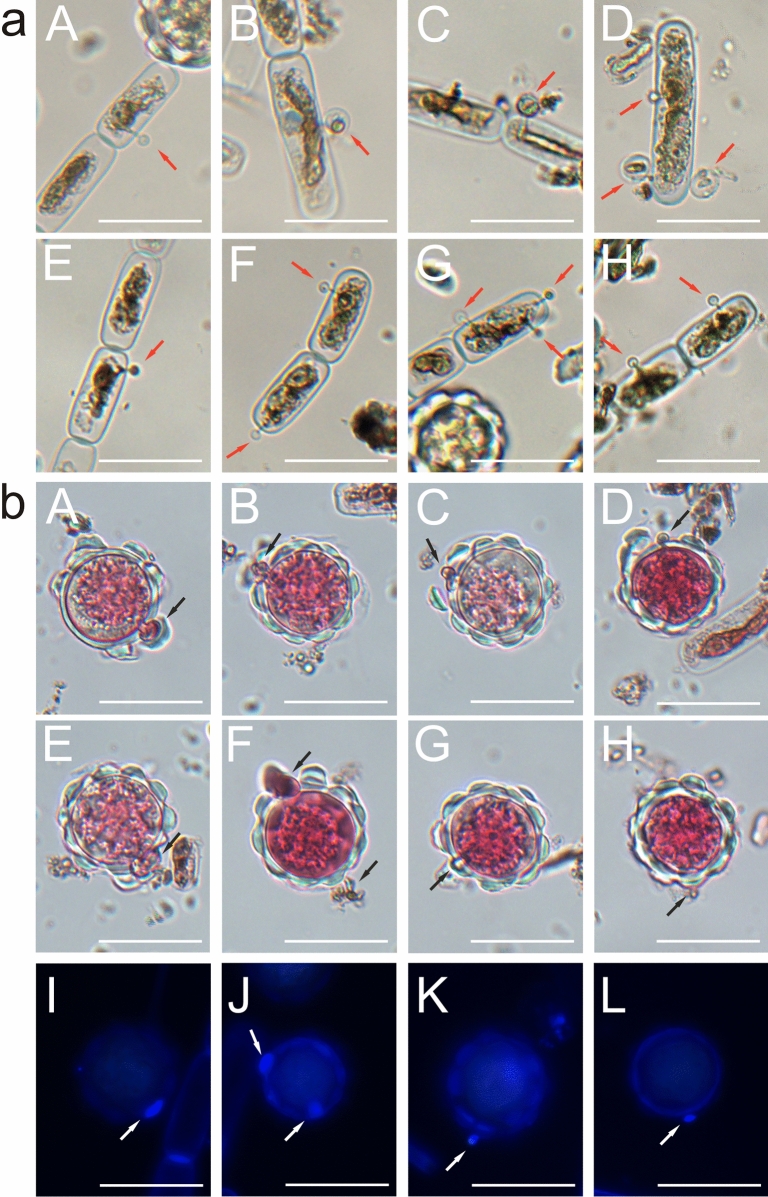
*A. nordenskioeldii* algae infected by fungi. **(a)**—the pictures were taken in brightfield microscopy; chytrids are visible as small circular structures attached to the algal cell walls (marked by red arrows) **(b)** hypnozygotes of *S. nivaloides* algae infected by the fungus after staining with propidium iodide observed in brightfield microscopy; the dye penetrated cells with a damaged wall and by binding with the genetic material. A–H-fungal cells growing in hypnozygotes; I–L hypnozygotes infected by fungi after staining with Calcofluor white; the chitin in the fungal cell wall fluoresces much more intensely than in the algal cells.

Figure 8a shows *A. nordenskioeldii* filamentous algae infected by the chytrid fungus visible as small circular structures attached to the algal cell walls (marked by red arrows). *A.* *nordenskioeldii* cells infected with the parasitic fungus were present only in the sample from site 2 and constituted approximately 25% of all cells of this species. Figure 8b shows Type 2 cells of *S. nivaloides* infected with chytrid fungi after staining with propidium iodide. The images were collected in brightfield microscopy. The dye penetrated cells with a damaged wall and stained the fungal cells by binding to the genetic material. It would have been difficult to observe the cells without the use of the dye. Images A-H in Fig. 8b show the fungal infection of Type 2 cells at various growth phases. Pictures A-D in Fig. 8b very clearly show a fungal cell (chytrid-like) penetrating an algal cell. Images C and D in Fig. 8b show the disintegration of the cell wall at the site of fungal growth. Pictures I-L in Fig. 8b show Type 2 cells infested by the parasite after staining with CW. The fungal chitin wall fluoresces much more intensely than the dimly glowing algal cells. No instances of fungus-infected Type 1 cell of *S. nivaloides* (cyst) were observed.

**Figure 9:**
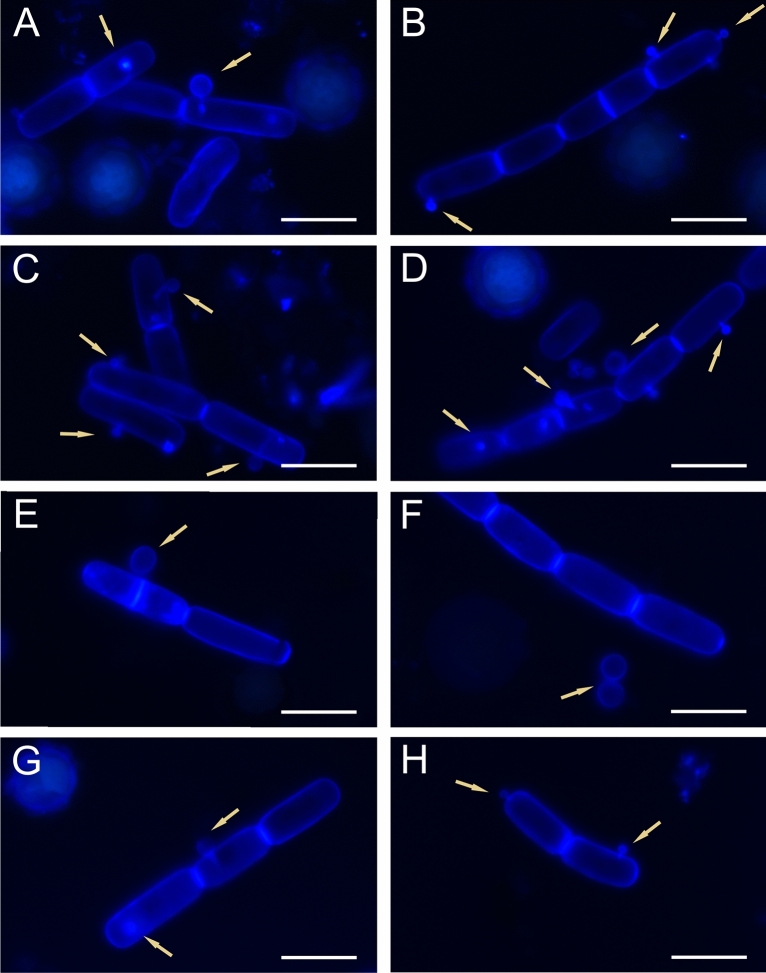
Microscopic images of *S. nivaloides* and *A. nordenskioeldii* cells from Upper Foxfonna Glacier (elevation 789 m) after staining with Calcofluor white. A–H—*A. nordenskioeldii* and parasitic fungus cells stained with Calcofluor white; fungal zoospores in various stages of development (marked with arrows). Images A–D, and G, H show glowing hypnozygotes. The scale bar corresponds to 20 µm.

Figure 9 illustrates algal cells stained with CW fluorochrome with blue fluorescence revealing the shape of *A. nordenskioeldii*. Small blue-glowing fungal cells were observed to protrude from the walls of the algae as well as mature sporangia detached from the algae cells (Fig. 9). After staining, it was observed that the dye specifically bound to chitin in the fungal cell wall (indicated by arrows in Fig. 9).

**Figure 10:**
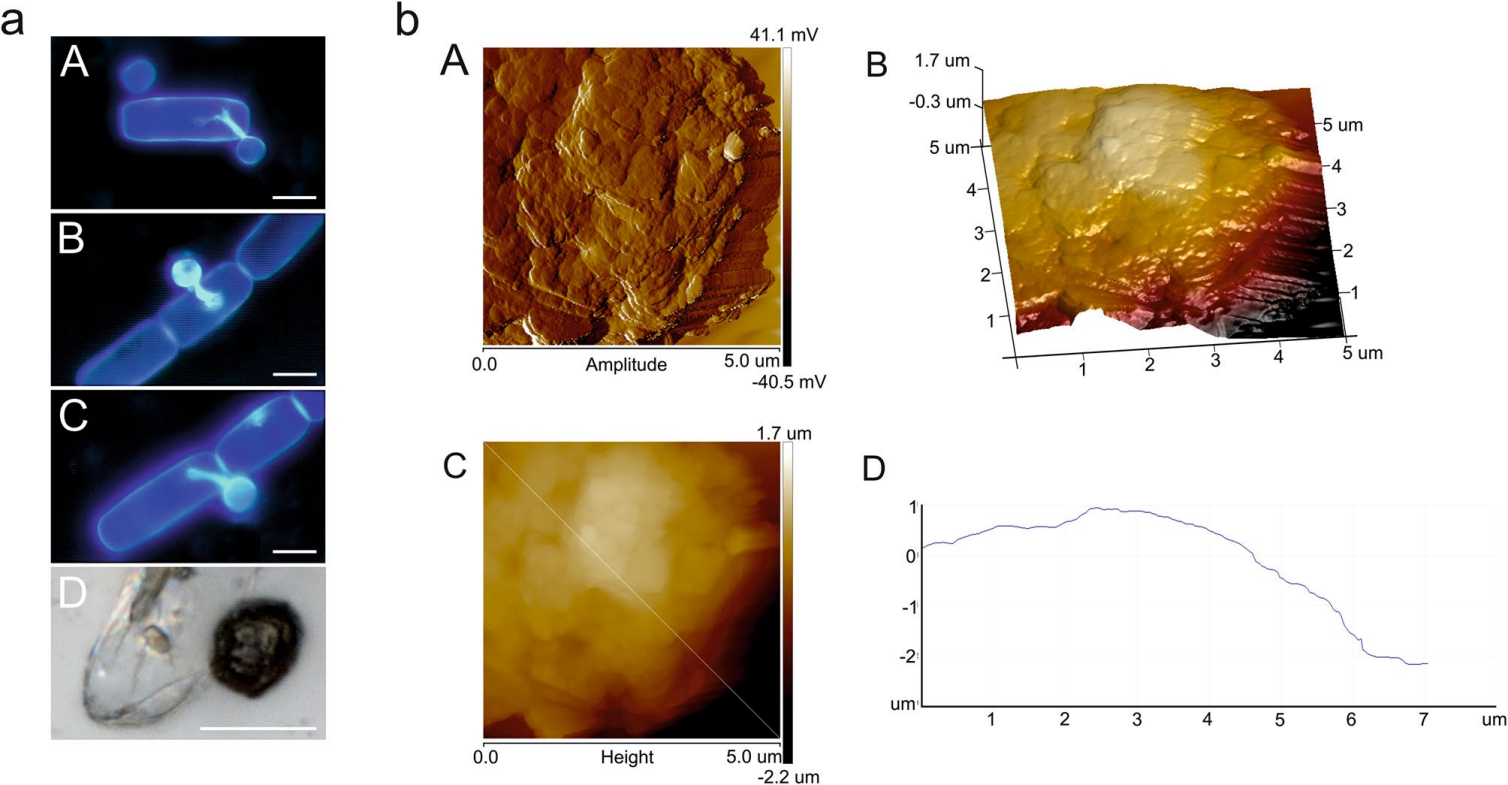
**(a)** Image of a parasitic fungal cell infecting an *A. nordenskioeldii* cell. A–C cell visualization after Calcofluor white staining. D—DIC image after SEM preparation. The scale bar corresponds to 5 µm, **(b)** AFM analysis of fungal cell from image. A—amplitude images of a fragment of the fungal cell surface; B—3D image—right panel; C—height image of the algal cell surface; D—height profile.

The fungal cell parasitizing *A. nordenskioeldii* was also examined with the AFM technique (Fig. 10a,b). Infected cells were stained with CW (Fig. 10aA–C) and visualized by DIC (Fig. 10aD). The cell in Fig. 10aD was analyzed by AFM, and the analysis showed a slightly folded cell surface (Fig. 10b).

### FTIR spectroscopy of algal samples

**Figure 11:**
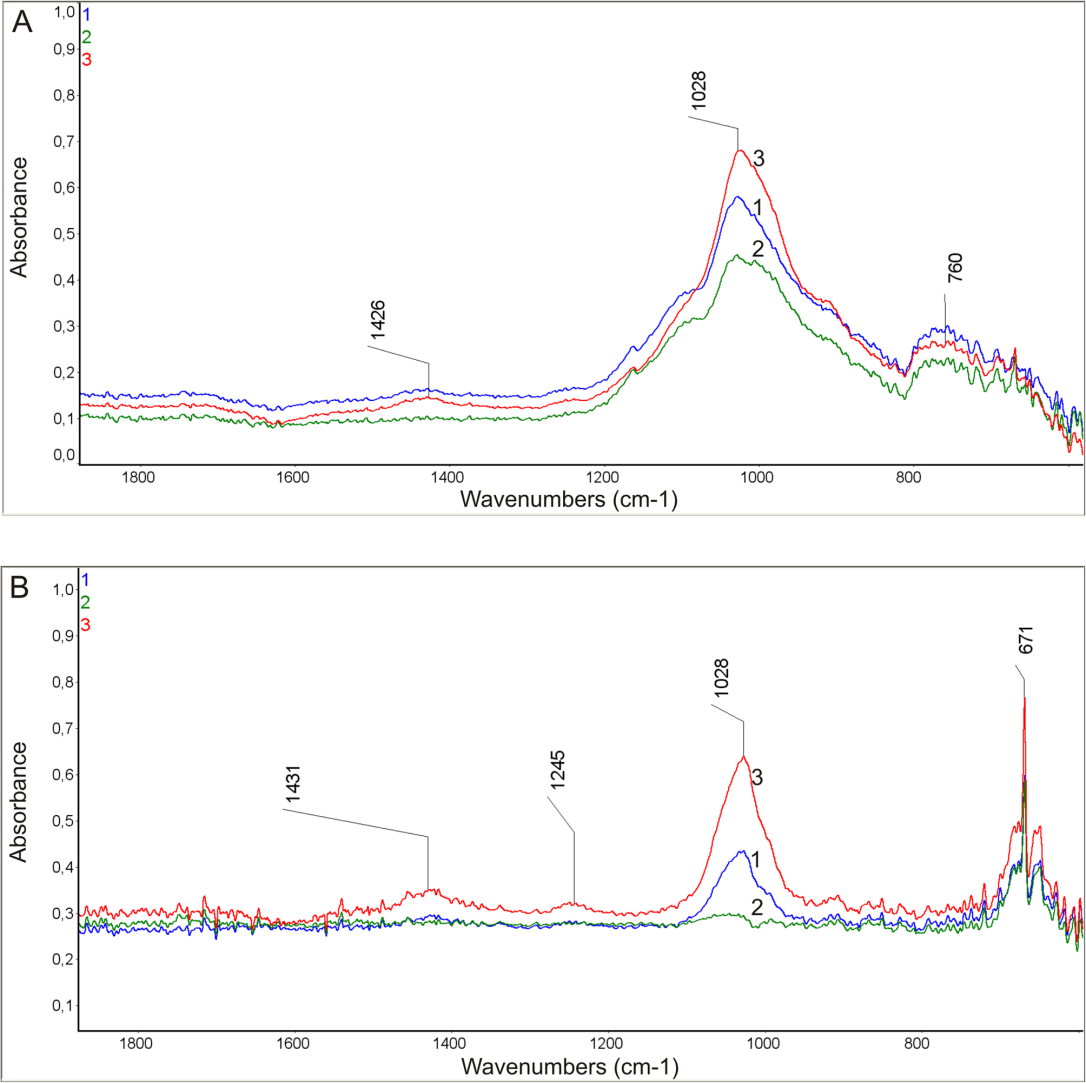
FTIR spectra made with the ATR technique from the solution: **(A)** algal suspension (pellet), **(B) **supernatant fraction (after removal of the fixative solution); sample 1—Longyearbreen Glacier, height 417 m; sample 2—Upper Foxfonna Glacier, height 789 m; sample 3—Lower Foxfonna Glacier, elevation 609 m.

Samples from the three sites in Svalbard were analyzed by FTIR spectroscopy. FTIR was employed to analyze the cell suspension and the secreted extracellular fraction. The analysis of both the cell suspension and the supernatant from the three-sample set showed the presence of a low intensity peak at position 1426 cm^−1^ for the cells and 1431 cm^−1^ for the supernatant, which corresponded to the bending vibration of the C-O groups (Fig. 11A). The spectrum of the supernatant fraction revealed a low intensity peak at 1245 cm^-1^ corresponding to the P=O stretching vibration derived from the phospholipids present in the algae. An intense peak at 1028 cm^−1^ was also observed for the FTIR-ATR spectrum. It corresponded to the stretching vibration of the O–C–O groups derived from pyranose rings (as shown by literature data), suggesting the presence of oligosaccharides in the tested samples. The least intense in this case was the spectrum peak of the sample from site 2 (Fig. 11B). The FTIR studies of both fractions showed the presence of peaks in the range of 800–600 cm^−1^ characteristic of the stretching vibrations of the C–Cl groups. During the analysis of the spectrum of the supernatant, the spectrum of the solvent was subtracted.

### SEM/energy dispersive X-ray spectroscopy (EDX)

The analysis of the supernatant samples of the algal cell suspension exhibited similar levels of most elements in sites 1 and 3, while the elemental composition of samples from site 2 differed significantly. The carbon content in all samples was similar and ranged from 47.85% to 50.05% (Table 2). The nitrogen content was approximately twofold higher in sample 1 (0.61%) than in sample 2 and 3. However, this small value did not exceed 1%. The percentage of such microelements as Mg, Cl, Ca, and Fe was higher in sample 2 than in the other tested samples, as shown in Table 2. These values were also below 1%. The high content of Al in the sample from site 2—5.41% is noteworthy. The content of this element in the samples from site 1 and 3 was 0.18% and 0.16%, respectively.

**Table 2:** Elemental composition of the supernatant fraction after separation from algal samples collected in different sites in Svalbard: 1—sample from Longyearbreen Glacier, elevation 417 m, 2—from Upper Foxfonna Glacier, elevation 789 m, 3—from Lower Foxfonna Glacier, elevation 609 m.

Element	Sample 1	Standard deviation	Sample 2	Standard deviation	Sample 3	Standard deviation
C	49.29	± 0.756	50.05	± 1.181	47.85	± 0.662
N	0.61	± 0.612	0.39	± 0.671	0.27	± 0.463
O	49.19	± 0.611	42.21	± 8.821	51.23	± 0.320
Na	0.20	± 0.038	0.67	± 0.149	0.14	± 0.012
Mg	0.09	± 0.018	0.47	± 0.519	0.05	± 0.008
Al	0.18	± 0.027	5.41	± 7.102	0.16	± 0.025
Si	0.23	± 0.038	0.27	± 0.095	0.12	± 0.019
S	0.04	± 0.013	0.05	± 0.015	0.02	± 0.005
Cl	0.03	± 0.013	0.13	± 0.045	0.02	± 0.000
K	0.05	± 0.019	0.15	± 0.044	0.05	± 0.000
Ca	0.06	± 0.027	0.09	± 0.029	0.03	± 0.005
Fe	0.04	± 0.026	0.07	± 0.043	0.03	± 0.000
P	0.00	–	0.01	± 0.012	0.04	± 0.038

### X-ray photoelectron spectroscopy (XPS) of algal samples

XPS analysis was performed to determine the elemental composition in the samples from sites 1 and 3. Spectra in a wide range of binding energies and spectra in a narrow range of binding energies characteristic for carbon, oxygen, nitrogen, and silicon were obtained. The results of the analyses are presented in Fig. S1A,B (Supplementary materials). The analyses showed the presence of carbon, nitrogen, oxygen, aluminum, silicon, calcium, and magnesium in the tested samples and a similar elemental composition of the samples from site 1 and 3. There were, however, slight differences in the content of oxygen and silicon in the samples from site 1 and 3. The different content of these elements in the tested samples may be related to the different amounts of rock material present in the collected samples.

To identify the bonds, present in the samples precisely, spectra were obtained in a narrow range of bonds for carbon, nitrogen, and silicon. The results of the analysis are presented in Tables 3, 4, and 5. The research showed the presence of aliphatic carbon atoms with C–C and C–H bonds. The tests also showed the presence of aromatic carbon atoms C=C, carboxyl groups O=C–OH, carbonyl groups C=O, and hydroxyl groups C–OH. These forms of carbon are found in oligosaccharides, polysaccharides, and lipids, which are components of algal cells. The XPS studies also revealed the presence of glycosidic bonds characteristic of cellulose, which is one of the components of the algal cell wall^34^. The XPS studies indicated the presence of bonds characteristic of proteins. The presence of N–H bonds and acid groups -COOH was demonstrated. The XPS studies carried out in the narrow range of binding energies for carbon and silicon showed the presence of CO_3_^2-^ and silica carbonates, which indicates the presence of rock material in the tested samples. The calcium and magnesium identified by the wide-range spectrum of aluminum may originate from the rock material as well.

**Table 3:** Elemental composition of snow alga samples: 1 (from Longyearbreen Glacier, elevation 417 m) and sample 3 (from Lower Foxfonna Glacier, elevation 609 m) determined by XPS.

Sample Identifier	Name	Position	Raw area	%At conc	% St.dev
1	C 1s	284.9	2495.200	77.1	1.36
	N 1s	399.9	56.958	1.0	0.75
	O 1s	532.4	1837.240	19.4	1.08
	Al 2p	73.9	12.242	0.7	0.55
	Si 2p	101.9	42.827	1.6	0.56
	Ca 2p	349.9	18.390	0.1	0.27
	Mg 1s	1304.4	28.826	0.1	0.10
3	C 1s	285.2	2430.200	76.4	1.50
	N 1s	400.2	45.201	0.8	1.04
	O 1s	532.7	1986.930	21.3	0.98
	Al 2p	74.7	5.948	0.4	0.80
	Si 2p	102.7	18.176	0.7	0.71
	Ca 2p	349.2	37.187	0.2	0.39
	Mg 1s	1304.7	69.484	0.2	0.16

**Table 4:** Elemental composition of sample 1 (from Longyearbreen Glacier, elevation 417 m) with the identification of characteristic chemical bonds^35–38^ determined by XPS.

Name	Position	Raw area	%At conc	Chemical bonds	Groups
C 1s A	285.00	193.740	34.0	C–C	Aliphatic carbon
C 1s B	284.50	46.561	8.2	C=C	Aromatic carbon
C 1s C	285.55	143.596	25.2	C–H	Aliphatic carbon
C 1s D	286.52	46.986	8.3	C–OH	Hydroxyl groups
C 1s E	287.08	38.800	6.8	C–O–C	Epoxy groups
C 1s F	287.63	3.619	0.6	C=O	Carbonyl groups
C 1s G	288.14	16.288	2.9	O=C–O–	Carboxyl groups
C 1s H	289.10	47.351	8.3	O = C–OH	Carboxyl groups
C 1s I	289.88	7.359	1.3	CO_3_^2−^	Carbonates
C 1s C-N	285.95	25.152	4.4	C–N	carbon–nitrogen bond
O 1s A	531.22	18.704	4.1	O^−2^	Metal oxides, hydroxides, carbonates
O 1s B	532.15	177.568	39.0	C–O–	Glycosidic groups
O 1s C	532.97	134.179	29.5	Si–O–Si, C–OH	Silica, hydroxyl groups (aliphatic)
O 1s D	533.71	111.801	24.6	Si–OH	Silica hydroxyl groups
O 1s E	534.53	11.738	2.6	O=C–O–	Carboxyl groups
O 1s F	535.37	1.000	0.2	C–OH, H_2_O/O_2_	Hydroxyl groups (aromatic), water/adsorbed oxygen
N 1s A	400.14	11.6698	91.73	−NH−	Amine
N 1s B	401.73	1.05185	8.27	−NH^+^−	Protonated amine
Si 2p 3/2 A	102.90	3.05771	100	Si–O	Aluminosilicate, silicates

**Table 5:** Elemental composition of sample 3 (from Lower Foxfonna Glacier, elevation, 609 m) with the identification of characteristic chemical bonds^35–38^ determined by XPS.

Name	Position	Raw area	%At conc	Chemical bonds	Groups
C 1s A	285.00	171.958	30.8	C–C	Aliphatic carbon
C 1s B	284.50	32.439	5.8	C=C	Aromatic carbon
C 1s C	285.55	134.456	24.1	C–H	Aliphatic carbon
C 1s D	286.52	53.899	9.7	C–OH	Hydroxyl groups
C 1s E	287.07	44.132	7.9	C–O–C	Epoxy groups
C 1s F	287.63	11.702	2.1	C=O	Carbonyl groups
C 1s G	288.14	20.628	3.7	O=C–O–	Carboxyl groups
C 1s H	289.10	45.854	8.2	O=C–OH	Carboxyl groups
C 1s I	289.97	11.347	2.0	CO_3_^2−^	Carbonates
C 1s C-N	285.95	31.516	5.7	C–N	Carbon–nitrogen bond
O 1s A	531.33	10.243	2.1	O^−2^	Metal oxides, hydroxides, carbonates
O 1s B	532.23	124.106	25.8	C–O–	Glycosidic groups
O 1s C	532.97	141.894	29.5	Si–O–Si, C–OH	Silica, hydroxyl groups (aliphatic)
O 1s D	533.72	154.388	32.1	Si–OH	Silica hydroxyl groups
O 1s E	534.43	42.700	8.9	O=C–O–	Carboxyl groups
O 1s F	535.33	7.566	1.6	C–OH, H_2_O/O_2_	Hydroxyl groups (aromatic), water/adsorbed oxygen
N 1s A	400.12	8.61974	80.6	−NH−	Amine
N 1s B	401.63	2.07451	19.4	−NH^+^−	Protonated amine
Si 2p 3/2 A	102.90	3.05771	100	Si–O	Aluminosilicate, silicates

## Discussion

The analysis of melting snow samples from three glacier sites in Svalbard showed the presence of two species of cryoflora: *S. nivaloides* and *A. nordenskioeldii*, which have been previously reported and determined for this region^39,40^. We found two types of cells: Type 1-cysts and Type- 2 hypnozygotes, which represented *S. nivaloides*^28,39,41^ (formerly *Chlamydomonas nivalis*), as shown by morphological observations. Our staining analysis revealed the presence of cellulose in the cell wall of the hypnozygotes. CW bound to the algal wall inducing blue fluorescence, while the AFM analysis of the hypnozygotes revealed a network of cellulose in the outer layer of the cell wall. The cysts revealed no fluorescence, which may be due to the presence of a polysaccharide envelope around the cell wall and the inability to bind the dye. In previous studies, *S. nivaloides* cysts from Alaska emitted weak blue fluorescence after CW staining^28^. The presence of cellulose in the walls of *Chlorophyceae* algae was discussed by Domozych^42^.

The DIC and SEM images of *A. nordenskioeldii* showed that the cell wall of this species is very delicate and shrinks easily. As described by Remias and collaborators^12^, the alga has an extremely thin and smooth cell wall with a thickness of only 90 nm. *A. nordenskioeldii* showed no fluorescence across all excitation wavelengths. Viable cells may show autofluorescence, but no fluorescence was observed after fixation. Remias et al.^12^ have indicated that the many brownish vacuoles most likely absorb excessive ultraviolet or visible light irradiation. Cell organelles, including chloroplasts, are thus protected against UV radiation, which can otherwise damage algal cell structures^43^. Staining of these cells with CW made it possible to observe the fluorescence of cellulose of the cell walls and clearly see the fungal cells parasitizing the algal cells. The chitin wall of the fungal cells glowed very intensely and thus allowed localization of the parasite, which is not always visible when employing brightfield microscopy.

Remias and collaborators^9^ reported colonization of *C. nivalis* (*S. nivaloides)* algal cells by the fungi. CW was used to stain the chitin cell wall of the parasitic fungi in our experiments. The cell wall of the sporangia and zoospores of these fungi glowed brightly under the fluorescence microscope. The identification of zoospores using CW was also used by Kagami et al.^18^ and Rad-Menéndez et al.^44^ After the growth period, a single protoplast turns into a sporangium and divides into zoospores. When a mobile naked zoospore encounters a sensitive algal cell, it attaches to the cell wall, surrounds it, and forms a thin germ tube that penetrates the algal wall. The parasitic fungi then produce enzymes that break down the algal cell contents. This content can be transferred to an encysted spore, which grows and transforms into a sporangium^45^. In our samples, the fungi clearly colonized the algal cells and led to their destruction. The DIC microscopic images showed hypnozygotes at different stages of decomposition. Some cells lost their cell wall integrity but retained the lobular structure, whereas others were completely digested by the enzymes of the parasite fungus.

The identification of fungal parasites such as chytrids is very difficult, as these fungi rarely possess species-specific morphological structures. The older classification was proposed by Kol (1968)^46^, who named this parasite of snow algae *Rhizophidium spharocarpum.* The relationship between the host and the parasite is sometimes so specific that the identification of the host consequently leads to identification of the parasitic fungus. Analyses of fungal communities associated with snow algae are motivated by the observation of high numbers of fungi found during microscopic imaging^46–49^. Research conducted by Brown et al.^45^ suggests that the presence of snow algae can act as an environmental filter^50^, presumably by providing physical substrates for attachment and nutritional subsidies for fungal growth. The abundance of these fungi in our study was surprising. Chytridiomycota fungi are more abundant and diverse than commonly believed. This diversity was observed by Freeman et al.^51^, highlighting chytrids were dominant in periglacial soils. Snow algae and snow fungi coexist due to their similar environmental preferences and tolerances^49^.

Kagami^18^ described one of *Chlamydomonas* parasites: a fungus from the genus *Rhizophydium* belonging to *Rhyzophydiales*. The morphology of the investigated parasite of snow algae is consistent with the morphology of chytrids. Zoospores have a spherical body 2–3 µm in size and stain with CW, which stains chitin. They can also appear both as attached to algae with a germ tube or as free-water cells. *Rhizophydiales* is an important group of chytrids. These fungi occur in soil and marine and freshwater habitats. *Rhizophydium* spp. can occur in these environments as parasites and organic matter-decomposing organisms^52^. They are parasites of many invertebrates, other chytrids, and algae and are important in the natural regulation of aquatic populations^24,53,54^. The processes of transformation of these organisms are still insufficiently studied. It is known that a zoospore must use its own stored reserves of lipids and glycogen when it is floating and will not attach to a suitable host or substrate. Our observations indicate that the zoospore is already equipped with a chitin wall when it is detached from the sporangium (Fig. 9).

Parasitic chytrid zoospores use light and chemical signals to locate hosts. For example, the zoospores of *Rhizophydium littoreum*, a parasite of marine green algae, exhibit positive phototaxis to blue light^55^. Through this mechanism, zoospores can enter the photic zone where they can find a suitable host. Both *R. littoreum* and *Batrachochytrium dendrobatidis* zoospores show chemotaxis to specific chemicals: sugars, proteins, and amino acids, which is a mechanism by which zoospores can detect signals specific to potential hosts^56,57^. Some observations indicate specific attachment of zoospores to a given host or species of algae. Certain chemical signals are involved in attracting zoospores^18^. Surface glycoproteins of zoospores were suggested as the potential site involved in encystment or recognition of environmental cues^58^. Some chytrid species were found to be positively attracted to both amino acids and carbohydrates^56^. This may indicate that chemical cues are involved in recognizing a potential algal host. Some microorganisms, such as Gram-negative *Sphingomonas* sp., show chemotaxis towards alginate and pectin polysaccharides. Chemotaxis towards macroparticles is less common than chemotaxis towards substances with low molecular weight such as amino or mono/oligopolysaccharides^59^.

It is surprising that, in our study, the fungal parasite did not appear to colonize *S. nivaloides* cysts. In contrast to cysts, *S. nivaloides* and *A. nordenskioeldii* hypnozygotes do not have a rich mucosa layer. Therefore, it is critical to examine the composition of the algal cell wall and compounds released into the environment to discern the chemical relationships that may play a role in the chemotaxis between host and parasite cells. FTIR and XPS spectroscopy were used to analyze chemical compounds released extracellularly by the algae. The FTIR analyses of the algal cell suspension and supernatant revealed the presence of bonds derived from polysaccharides, fatty acids, and amino acids. The polysaccharide-derived spectral peaks were much more intense and diverse than the lipid signals. The FTIR spectra suggest the presence of pyranose rings present in oligosaccharides. The highest intensity of the peaks was recorded in the samples from sites 1 and 3; in these samples, the cyst-type cells dominated. This may be related to the presence of a mucous polysaccharide sheath surrounding this type of cells and the release of compounds into the environment. Our preliminary studies have shown that the obtained polysaccharide fraction has antifungal activity. This may be supported by the fact that no cysts parasitized by chytrids were observed. We intend to address this interesting issue in future research.

The XPS analyses of the samples confirmed the above observations. The similarity of the XPS spectra of the samples from sites 1 and 3 suggests that the analyzed extracellular polysaccharide fraction can be derived from *S. nivaloides* cysts, which are present in large numbers. The elemental analysis of the supernatant of individual samples showed the highest oxygen content in samples from sites 1 and 3, which corresponds to the larger amount of oxygen from carboxyl groups in polysaccharides. The oxygen content in these samples was at a similar level. The elemental composition of the sample from site 3 was rich in various microelements and was characterized by particularly high content of aluminum, which indicates the composition of the mineral dust.

Fungal infections of host algae are not possible to develop below a certain threshold value of the cell host density, irrespective of temperature and light conditions^60^. There are also hypotheses that parasite cells infect large-sized host cells. As reported by Sime-Ngando^52^, the host abundance as well as the host cell size and biomass probably establish the threshold limit for the critical prevalence of fungal infection. The conducted analyses provided better resolution of the morphology of snow algal cells and their fungal parasites assessed using microscopic and spectroscopic methods. They also focus further research on the search for chemical and physical relationships in the host-parasite relationship, and the potential scale of impacts for the carbon and nutrient budgets following fungal parasitism on glacial and snow algae. The rich variety of fungal species in Svalbard^1^ offers great opportunities to discover new relationships between microorganisms in this Arctic Archipelago.

## Supplementary Information

Supplementary Figure S1.

Supplementary Legends.
