# Supplementary figures and images for: Morphological and spectroscopic analysis of snow and glacier algae and their parasitic fungi on different glaciers of Svalbard
Source: Sci Rep. 2021 Nov 8;11:21785. doi: 10.1038/s41598-021-01211-8 (PMC8575968; doi:10.1038/s41598-021-01211-8)

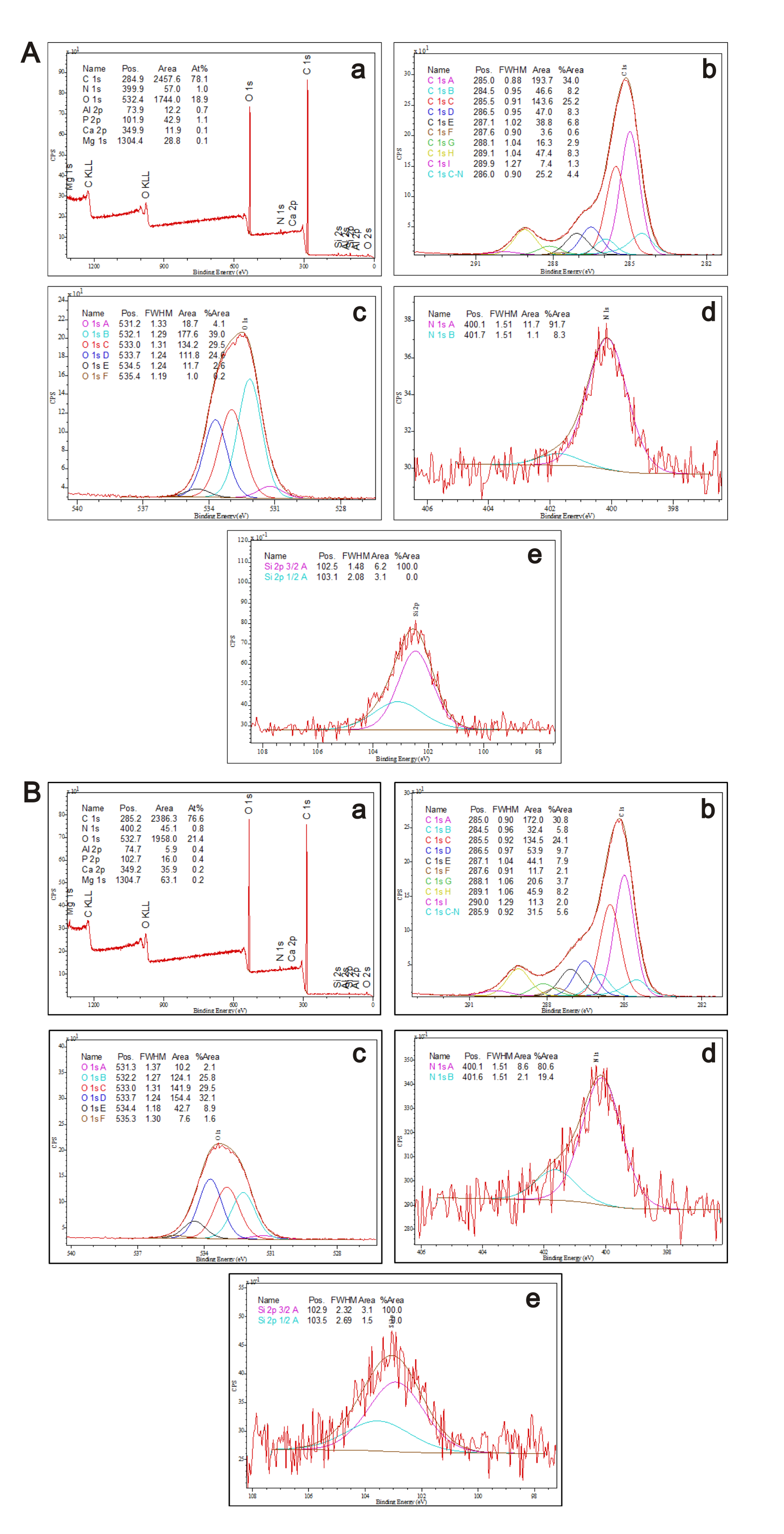

Supplement: Supplementary file 1 — Supplementary Figure S1. [file 41598_2021_1211_MOESM1_ESM.tif]
