# Supplementary material for: Morphological and spectroscopic analysis of snow and glacier algae and their parasitic fungi on different glaciers of Svalbard
Source: Sci Rep. 2021 Nov 8;11:21785. doi: 10.1038/s41598-021-01211-8 (PMC8575968; doi:10.1038/s41598-021-01211-8)
Supplement: Supplementary file 2 — Supplementary Legends. [file 41598_2021_1211_MOESM2_ESM.docx]

**Supplementary materials**

**Fig. S1.** XPS spectrum of the algal supernatant fraction (after removal of the fixative solution). XPS spectra obtained for sample 1 (Longyear Glacier) and 3 (Foxfonna Glacier, 696 m): a) spectrum in a wide binding energy range, b) spectrum in a narrow energy range characteristic for carbon, c) spectrum in a narrow energy range characteristic for oxygen, d) spectrum in a narrow energy range characteristic for nitrogen, e) spectrum in the narrow energy range characteristic for silicon.
